# The relationship between blood vitamin A levels and diabetic retinopathy: a population-based study

**DOI:** 10.1038/s41598-023-49937-x

**Published:** 2024-01-04

**Authors:** Yu-Jin Choi, Jin-Woo Kwon, Donghyun Jee

**Affiliations:** 1 Department of Ophthalmology and Visual Science, College of Medicine, The Catholic University of Korea, Seoul, Korea; 2 Department of Ophthalmology and Visual Science, St. Vincent’s Hospital, Jungbu-daero 93, Paldal-gu, Suwon, 16247 Korea

**Keywords:** Health care, Medical research

## Abstract

We assessed the relationship between blood vitamin A levels and the risk of diabetic retinopathy. The study was population-based epidemiological study for 11,727 participants aged 40 or older who participated in the Korean National Health and Nutrition Examination Survey. Vitamin A in the blood was classified into quartiles. Diabetic retinopathy was diagnosed by the Early Treatment for Diabetic Retinopathy Study. After adjusting confounding variables such as age, sex, smoking, cholesterol, diabetes prevalence period, glycated hemoglobin levels, and high blood pressure, the odd ratio (OR) of vitamin A at quartile level 4 for diabetic retinopathy was 0.32 (95% confidence interval [CI], 0.14–0.72, P for trend < 0.001). In male, the OR of quartile 3 level vitamin A for diabetic retinopathy was 0.11 (95% CI, 0.01–0.69, P for trend = 0.010). In adults under the age of 60, the OR of vitamin A at quartile level 3 for diabetic retinopathy was 0.10. (95% CI, 0.03–0.29, P for trend < 0.001). Serum vitamin A high levels are associated with low risk of diabetic retinopathy. Particularly, there is a more effective relationship in male and adults under the age of 60.

## Introduction

In the case of diabetes, complications can occur in various parts of the body, among which complications that may happen in the eyes are diabetic retinopathy. Diabetic retinopathy is caused by angiogenesis, inflammation, and the formation of fibrous connective tissue^1–3^. Diabetic retinopathy can cause blindness such as vitreous bleeding, diabetic macular edema, neovascular glaucoma, and fibrosis as inflammatory molecules are generated and angiogenesis occurs during the disease^4^. In South Korea, 20.9% of diabetic patients with a disease duration of 6 to 10 years and 66.7% of patients with a disease duration of 15 years or more suffer from diabetic retinopathy. Diabetic retinopathy itself does not increase the mortality rate, but if diabetic patients lose their eyesight, their quality of life decreases and healthcare expenditure increases^5^.

Vitamin A is essential for maintaining immune system, cell differentiation and maintaining vision^6^. Vitamin A has a positive effect on inhibiting angiogenesis, inflammation, and fibrosis^7,8^. Vitamin A is thought to play an important role in the process of angiogenesis, inflammation, and fibrosis, which are the causes and mechanisms of diabetic retinopathy. Vitamin A inhibits angiogenesis by inhibiting vascular endothelial growth factor. And vitamin A works to suppress inflammation by reducing cytokines in the blood flow that increase during the inflammation process. During inflammation, cytokines interleukin-6 and interleukin-1 increase, which induce the synthesis of other acute inflammatory biomarkers as initiators of acute inflammation^9^. Vitamin A also takes the function of inhibiting fibrosis. Fibrosis builds on the activation of hyaluronidase, which involves increased permeability of the basal membrane and vascular wall, which in turn leads to exudation of lymph, white blood cells, fibrinogen and macrophages. The macrophages confined to produced fibrin matrix are differentiated into the fibroblasts that produce collagen actively^10^. Vitamin A has an action of inhibiting fibrosis, and in animal models, Immunohistochemistry has shown that improved expression of all three fibrosis markers has been reduced due to vitamin A administration^11^.

Considering the effect of vitamin A, it is thought that vitamin A will take an important role in the process of angiogenesis, inflammation, and fibrosis, the cause and mechanism of diabetic retinopathy. There are several previous studies showing a relationship between vitamin A and diabetic retinopathy. In a study by Rostamkhani et al. for 60 diabetic patients, increasing serum vitamin A levels reduced the risk of diabetic retinopathy by 31.1%^12^. We previously conducted an epidemiological study using dietary vitamin A and reported that the relationship with the risk of diabetic retinopathy did not show statistical significance, highlighting the importance of methodology^13^. However, studies using serum vitamin A rather than dietary vitamin A are rare, and in particular, studies using serum vitamin A have small samples. Therefore, we conducted the large epidemiological study to evaluate the risk of diabetic retinopathy using serum vitamin A. This study was accompanied using Korea National Health and Nutrition Examination Survey (KNHANES) data from adults aged 40 or older representing Korea to evaluate the relationship between diabetic retinopathy and vitamin A, which has the function of inhibiting angiogenesis, anti-inflammatory effects, and antifibrotic effects.

## Results

The clinical and demographic characteristics of 260 subjects who participated in the study were shown in Table 1 according to status of diabetic retinopathy. Compared to those without diabetic retinopathy, subjects with diabetic retinopathy had higher systolic blood pressure (P = 0.004), higher fasting blood sugar (P < 0.001), higher glycated hemoglobin levels (P < 0.001), longer diabetic prevalence periods (P < 0.001), and lower cholesterol levels (P = 0.006). And there were differences in diabetic retinopathy status according to the degree of diabetes prevalence (P < 0.001) and the degree of vitamin A level in the blood (P = 0.042). Of the overall 260 subjects, the group without diabetic retinopathy had 211 subjects and the group with diabetic retinopathy had 49 subjects. The distribution of diabetic retinopathy, according to the quartile of blood vitamin A in the participants, is shown in Table 2 along with estimated value and unweighted frequency.

**Table 1:** Clinical and demographic characteristics, according to diabetic retinopathy status, as reported in the Korean National Health and Nutrition Examination Survey 2016–2018. Value is described as weighted means or weighted frequency (%) with standard errors. *DR* diabetic retinopathy, *HbA1c* glycated hemoglobin, *BMI* body mass index.

Characteristics	Without DR	Any DR	*P*	Participants
Male (%)	57.9 (1.4)	59.1 (2.7)	0.729	58.2 (1.2)
Age (years)	61.3 (0.2)	61.5 (0.5)	0.791	61.4 (0.3)
Systolic blood pressure (mmHg)	125.0 (0.3)	128.3 (1.0)	0.004	126.6 (0.5)
Diastolic blood pressure (mmHg)	76.2 (0.3)	75.7 (0.5)	0.458	75.9 (0.3)
Fasting glucose (mg/dL)	135.1 (0.9)	155.8 (3.7)	< 0.001	145.4 (1.8)
HbA1c (%)	6.93 (0.0)	7.73 (0.1)	< 0.001	7.33 (0.0)
Diabetes severity			< 0.001	
Normal	1.0 (0.1)	0.0 (0.0)		0.8 (0.1)
Fasting blood sugar disorder	10.5 (0.7)	2.0 (0.3)		8.8 (0.6)
Diabetes	88.5 (0.7)	98.0 (0.3)		90.4 (0.6)
Diabetic duration (years)	8.0 (0.2)	11.9 (0.5)	< 0.001	9.9 (0.3)
Total cholesterol (mg/dL)	180.1 (1.2)	170.2 (3.1)	0.006	175.2 (1.7)
Triglyceride (mg/dL)	180.0 (3.8)	167.2 (6.5)	0.098	173.6 (3.7)
Vitamin A (mg/L)	0.63 (0.0)	0.63 (0.0)	0.973	0.63 (0.0)
Vitamin A (%)			0.042	
Vit A < 0.41	8.2 (1.4)	14.4 (4.1)		9.4 (1.4)
0.41 ≤ Vit A < 0.51	19.8 (2.2)	22.6 (4.3)		20.3 (2.0)
0.51 ≤ Vit A < 0.64	29.6 (2.1)	17.1 (2.6)		27.2 (1.6)
Vita A ≥ 0.64	42.4 (2.4)	46.0 (5.0)		43.1 (2.2)
Hypertension (%)			0.276	
Normal	19.8 (1.0)	19.0 (3.2)		19.7 (1.1)
Prehypertension	19.7 (1.1)	15.9 (1.6)		18.9 (0.9)
Hypertension	60.5 (1.4)	65.1 (2.9)		61.4 (1.3)
Body mass index (kg/m^2^)			0.240	
BMI < 18.5	0.9 (0.3)	0.9 (0.7)		0.9 (0.3)
18.5 ≤ BMI < 23.0	27.0 (1.1)	24.6 (2.9)		26.5 (1.2)
23.0 ≤ BMI < 25.0	21.9 (0.9)	27.4 (2.2)		23.0 (0.8)
25.0 ≤ BMI < 30.0	40.1 (1.3)	39.4 (2.7)		40.0 (1.2)
30.0 ≤ BMI < 35.0	9.8 (0.8)	7.0 (1.1)		9.2 (0.7)
BMI ≥ 35	0.4 (0.1)	0.7 (0.5)		0.4 (0.1)
Hyperlipidemia (%)	45.5 (1.4)	42.0 (3.0)	0.299	44.8 (1.2)
Hypertriglyceridemia (%)	26.7 (1.4)	20.4 (2.7)	0.057	25.5 (1.2)
Smoking status				
Never (%)	47.9 (1.4)	46.1 (2.7)		47.6 (1.3)
Former (%)	32.5 (1.3)	30.6 (2.4)		32.1 (1.2)
Current (%)	19.5 (0.9)	23.2 (2.8)		20.3 (1.0)

**Table 2:** Distribution of diabetic retinopathy according to quartile levels of blood vitamin A in representative Korean adults aged 40 years or older. Value is described as estimated value (%) or standard error (%) or unweighted frequency. *DR* diabetic retinopathy.

Quartile blood vitamin A level (mg/L)	Diabetic retinopathy status		
	Without DR	DR	Total
Quartile level 1 (< 0.41)			
Estimated value	73.1%	26.9%	100.0%
Standard error	4.3%	4.3%	0.0%
Unweighted frequency	49	15	64
Quartile level 2 (0.41–0.51)			
Estimated value	90.4%	9.6%	100.0%
Standard error	2.2%	2.2%	0.0%
Unweighted frequency	58	10	68
Quartile level 3 (0.51–0.64)			
Estimated value	79.4%	20.6%	100.0%
Standard error	3.6%	3.6%	0.0%
Unweighted frequency	52	13	65
Quartile level 4 (≥ 0.64)			
Estimated value	80.2%	19.8%	100.0%
Standard error	2.7%	2.7%	0.0%
Unweighted frequency	52	11	63
Total			
Estimated value	80.7%	19.3%	100.0%
Standard error	1.8%	1.8%	0.0%
Unweighted frequency	211	49	260

Blood vitamin A levels were associated with diabetic retinopathy. After adjusting confounding variables, people with blood vitamin A quartile 3 levels had an odd ratio (OR) of 0.17 compared to those with the lowest quartile level (95% CI, 0.08–0.42, P for trend < 0.001). In people with blood vitamin A quartile 4 levels, the OR after adjusting all seven confounding variables was 0.32 compared to those with the lowest quartile level (95% CI, 0.14–0.72, P for trend < 0.001) (Table 3).

**Table 3:** Adjusted odds ratio of diabetic retinopathy stratified according to quartile levels of blood vitamin A in representative Korean adults aged 40 years or older. Value is described as odd ratio (95% confidence intervals). Model 1: adjusted for age and sex. Model 2: adjusted for age, sex, smoking, cholesterol, diabetes duration, glycated hemoglobin, and hypertension.

Quartile blood vitamin A level (mg/L)	Crude	Model 1	Model 2
Quartile level 1 (< 0.41)	1.0 (reference)	1.0 (reference)	1.0 (reference)
Quartile level 2 **(**0.41–0.51)	0.65 (0.26–1.60)	0.55 (0.20–1.51)	0.50 (0.20–1.24)
Quartile level 3 (0.51–0.64)	0.32 (0.14–0.74)	0.28 (0.11–0.68)	0.17 (0.08–0.42)
Quartile level 4 (≥ 0.64)	0.62 (0.26–1.42)	0.46 (0.18–1.17)	0.32 (0.14–0.72)
P for trend	0.014	0.016	0.001

For each sex, the relationship with the risk of diabetic retinopathy according to the degree of blood vitamin A level was shown in Table 4. In male, the OR of blood vitamin A for diabetic retinopathy after adjusting confounding variables was 0.11 (95% CI, 0.01–0.69, P for trend = 0.010). However, in female, there was no statistical difference between diabetic retinopathy and blood vitamin A levels after adjusting confounding variables.

**Table 4:** Odds ratio of diabetic retinopathy in male and female stratified according to the quartile category of vitamin A in the blood in representative Korean adults aged 40 years or older, after adjusting for age, sex, smoking, cholesterol, diabetes duration, glycated hemoglobin, and hypertension. Value is described as odds ratio (95% confidence intervals).

Quartile blood vitamin A level (mg/L)	Odds ratio
Male	
Quartile level 1 (< 0.41)	1.0 (reference)
Quartile level 2 **(**0.41–0.51)	0.40 (0.85–1.89)
Quartile level 3 (0.51–0.64)	0.11 (0.01–0.69)
Quartile level 4 (≥ 0.64)	0.27 (0.05–1.39)
P for trend	0.010
Female	
Quartile level 1 (< 0.41)	1.0 (reference)
Quartile level 2 (0.41–0.51)	0.82 (0.25–2.65)
Quartile level 3 (0.51–0.64)	0.47 (0.22–1.01)
Quartile level 4 (≥ 0.64)	0.42 (0.14–1.27)
P for trend	0.277

Regarding age, the relationship with the risk of diabetic retinopathy according to the degree of blood vitamin A level was shown in Table 5. For those under 60, in people with blood vitamin A quartile 3 levels, the OR for diabetic retinopathy was 0.10 (95% CI, 0.03–0.29, P for trend < 0.001) compared to those with the lowest quartile level after adjusting confounding variables. For those over 60 years of age, in people with blood vitamin A quartile 3, the OR for diabetic retinopathy was 0.21(95% CI, 0.07–0.59, P for trend = 0.045), compared to those who had the lowest quartile level after correcting confounding variables. For those aged 60 and older, the OR after adjusting all seven confounding variables was 0.20 (95% CI, 0.06–0.68, P for trend = 0.045) compared to those with the lowest quartile level in people with blood vitamin A quartile 4 levels.

**Table 5:** Odds ratio of diabetic retinopathy in older and younger adults stratified according to the quartile category of vitamin A in the blood in representative Korean adults aged 40 years or older, after adjusting for age, sex, smoking, cholesterol, diabetes duration, glycated hemoglobin, and hypertension. Value is described as odds ratio (95% confidence intervals).

Quartile blood vitamin A level (mg/L)	Odds ratio
Age < 60	
Quartile level 1 (< 0.41)	1.0 (reference)
Quartile level 2 **(**0.41–0.51)	0.35 (0.02–5.28)
Quartile level 3 (0.51–0.64)	0.10 (0.03–0.29)
Quartile level 4 (≥ 0.64)	0.23 (0.05–1.01)
P for trend	< 0.001
Age ≥ 60	
Quartile level 1 (< 0.41)	1.0 (reference)
Quartile level 2 **(**0.41–0.51)	0.34 (0.11–1.06)
Quartile level 3 (0.51–0.64)	0.21 (0.07–0.59)
Quartile level 4 (≥ 0.64)	0.20 (0.06–0.68)
P for trend	0.045

## Discussion

Our study found that vitamin A was associated with lowering the risk of diabetic retinopathy. Particularly, it was found that vitamin A was more associated with the low risk of diabetic retinopathy in males and younger under 60. And when blood vitamin A level was above 0.51 and below 0.64, it was found that the risk of developing diabetic retinopathy was reduced compared to other vitamin A concentrations in the blood. This study is the first study to examine the relationship between serum vitamin A and the risk of diabetic retinopathy.

The reason for this result that vitamin A reduces the risk of diabetic retinopathy can be explained by the mechanism of developing diabetic retinopathy and the mechanism of action for vitamin A. The mechanisms for developing diabetic retinopathy include angiogenesis, inflammatory reactions, and fibrosis^14,15^. With these pathogenesis**,** diabetic retinopathy develops complications such as non-proliferative diabetic retinopathy, proliferative diabetic retinopathy, vitreous hemorrhage, and traction retinopathy^16,17^. On the contrary, vitamin A has anti-angiogenesis, anti-inflammatory reactions, and anti-fibrotic functions, which are contrary to the onset of diabetic retinopathy^18,19^. Vitamin A is a powerful regulator of cell differentiation and proliferation and acts as an immunomodulatory and antiangiogenic activity^20–22^. Vitamin A is one of the representative antioxidants and acts as an anti-inflammatory to protect the body in response to inflammation in the body by removing active oxygen and improving immune function in the body^23,24^. Vitamin A has anti-fibrotic effects, so studies have been conducted on the treatment of vitamin A-binding drugs in diseases such as pulmonary fibrosis, liver fibrosis, and skin fibrosis. And vitamin A is also expected to have anti-fibrotic effects in the retina of our eyes^25–28^. For these reasons, vitamin A can be explained to decrease the risk of diabetic retinopathy.

In this study, vitamin A was found to be associated with a low risk of diabetic retinopathy, especially in men. These results can be explained by biological, social behavior, and cultural differences between men and women. According to one study, the average level of each biomarker varied significantly by gender at birth, cardiac metabolism biomarkers were higher in men, and inflammation and neuroendocrine biomarkers were higher in women. However, over time, the biomarker characteristics of gender were mediated, attenuated, or interacted with each other according to socio-environmental differences^29^.

Our study found that higher levels of blood vitamin A were associated with a lower risk of diabetic retinopathy especially in people younger than 60, compared to people age 60 and older**.** Vitamin A acts in the visual system by transformation between all-trans-retinal and 11-cis-retinal^30^. In the case of old age, metabolic activity in our body decreases as we age, which can be explained as the reason why blood vitamin A levels have a greater effect on lowering the risk of diabetic retinopathy in young people under the age of 60. It is thought that with age, the function of mitochondria decreases and the sensitivity of vitamin A to the retina decreases, resulting in these results^31,32^. The effect of vitamin A may be greater in young people due to the greater metabolic activity of photoreceptors.

The level 3 of vitamin A was more effective to reduce the risk of diabetic retinopathy than the level 4. And when blood vitamin A level was between 0.51 and 0.64, it was found that the risk of developing diabetic retinopathy was reduced compared to other vitamin A concentrations in the blood. Considering the cutting point of vitamin A was 0.64, vitamin A levels above 0.64 becomes less effective to reduce the risk of diabetic retinopathy than level 3. The normal range of vitamin A is from 0.3 to 0.6. The upper limit of normal vitamin A level in blood is 0.6. So excessive vitamin A above normal limit is less effective to reduce risk. Although exact reason is unclear, vitamin A is fat soluble, so excessive vitamin A is not good for diabetic retinopathy.

There are several studies on vitamin A and diabetic retinopathy. Ruamviboonsuk et al. reported that serum vitamin A levels were lower in diabetic participants without retinopathy than in the DR group. Patients with DR had significantly lower serum vitamin A levels than control (P = 0.01). Higher serum vitamin A levels reduced the risk of DR development by 31.1% (P = 0.007)^33^. Rostamkhani et al. reported that the low concentration of vitamin A in the blood was related to the severity of diabetic retinopathy, followed by the group without diabetic retinopathy, the group with non-proliferative diabetic retinopathy, and the group with proliferative diabetic retinopathy^12^. Zhang et al. reported that dietary vitamin A intake was lower in the group with diabetic retinopathy than in the group without diabetic retinopathy^34^. In the past, we conducted a study on 1948 adults over the age of 40 representing Korea on the risk of dietary vitamin A levels and diabetic retinopathy^13^. However, we did not find evidence of association between high levels of dietary vitamin A and low risk of diabetic retinopathy. Our previous study did not find evidence of the relationship between vitamin A and diabetic retinopathy through dietary information. This does not mean that diabetic retinopathy is not related to dietary vitamin A, but measuring the amount of dietary vitamin A means that there is a limitation. Vitamin A is a fat-soluble vitamin, and if people have conditions such as small intestine disorders, chronic absorption disorders, and decreased pancreatic function, the absorption rate can vary^35^. Also, dietary vitamins A is absorbed into the blood and act in different forms when they actually act on the retina^30^. Therefore, we think measuring vitamin A levels in the blood type rather than the dietary type is more suitable for evaluating the relationship between diabetic retinopathy and vitamin A. This means that identifying association through blood sampling is a more accurate and effective way to see and interpret the results. Through this study, we were able to find that high levels of blood vitamin A were associated with a low risk of diabetic retinopathy.

Diabetic patients with diabetic retinopathy need health policies or health education supplements. As diabetic retinopathy worsens, quality of life decreases and vision decreases, which can lead to dangerous accidents, and healthcare expenditure rises^36^. Therefore, health policies or health education supplements for diabetes control and diet control are needed for patients with diabetic retinopathy. Ultimately, education such as diet control that can increase vitamin A, as revealed through this study, is needed. Through health policies and health education, we must ensure that patients can consume vitamin A and create an environment in which vitamin A can be synthesized well.

The strength of this study is that the study was conducted on a large number of people through random sampling of statistical multi-level clusters. Considering the formula for calculating the P value, the statistical significance increases when the effect is large or the sample size is large. Conversely, if the number of samples is small, statistical significance may decrease. We selected 260 subjects suitable for the study from a total of 11,727 subjects, and the results showed statistical significance. The fact that a statistically significant result was obtained with a small number of samples actually has much greater statistical significance. In previous studies, most were case–control studies. This may have selection bias. Our study is a population-based study, which is significance compared to previous studies. We conducted study targeting people with diabetes and diabetic retinopathy among all people without any manipulation of sampling. In addition, by measuring blood vitamin A levels, not diet, more scientific and accurate results could be achieved. Our research has some limitations. This study is the results of research in South Korea and cannot represent other regions. There may be different results for other ethnic groups or regions, so further studies are needed. Our study is a cross-sectional study. The microvascular complication such as diabetic retinopathy are result from the long duration of metabolic control, several years before the onset of diabetic retinopathy as previous report in Asian population^37^. So, the cross-sectional vitamin A level might not represent the vitamin throughout several year level in the body. We need large sample size cohort study. The model that was adjusted only recent glycemia, blood pressure should interpret in cautions. Since it is a cross-sectional study, it can be seen that there is a relationship with each other, but it was difficult to confirm the information according to the cause and effect, that is, which comes first. The use of prospective health survey data remains necessity. The results are interesting and motivate bigger studies to confirm the observation. Therefore, further studies in the future are needed to supplement these limitations.

In conclusion, a high level of vitamin A in the blood is associated with a low risk of diabetic retinopathy. In particular, there is a more effective relationship between male and young people under the age of 60. Increasing vitamin A in the blood to reduce the incidence of diabetic retinopathy is thought to help prevent diabetic retinopathy. Therefore, it is necessary to create a good environment for consuming vitamin A to increase vitamin A or synthesizing it on its own. Further research on future care, health policies, or health education for patients vulnerable to diabetic retinopathy is thought to be needed in the future.

## Methods

**Figure 1:**
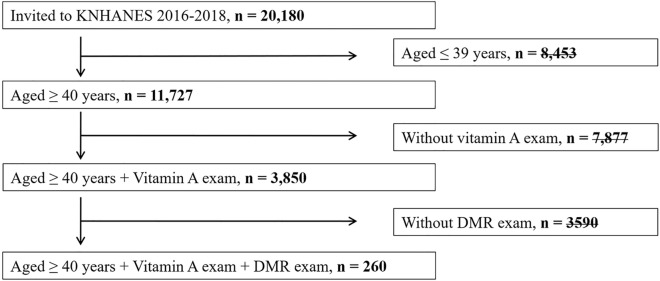
Flow chart presenting the selection of study participants.

This study was conducted using data from KNHANES from 2016 to 2018. KNHANES is a population-based data survey using statistical, multi-level, and clustered sampling methods representing Korea. All participants provided informed consent for the use of their clinical records. From 2016 to 2018, the total number of people who participated in KNHANES was 20,180. Of these, 8453 people under the age of 39 were excluded. Of the 11,727 participants aged 40 or older, 7877 people who did not take blood vitamin A tests and 3590 who did not take fundus imaging tests were excluded from the study. Finally, 260 participants who performed blood vitamin A tests and fundus imaging tests were included in study (Fig. 1). Because the prevalence of diabetic retinopathy increases in people over 40 years of age, many epidemiological studies in various countries target this age demographic, when determining the risk of diabetic retinopathy^38–44^. We also targeted people over 40 years of age. The study was approved by the Institutional Review Board of the Catholic University of Seoul in Korea and all methods were carried out in accordance with the principles of the Helsinki Declaration (IRB number: VC22ZESI0175).

Health interviews including age, sex, body mass index, and smoking status were conducted for participants. The body mass index was classified into six stages. The cutting points were 18.5, 23.0, 25.0, 30.0, and 35.0. Smoking status was classified into three categories: non-smoker, past smoker, and current smoker. Blood pressure was taken by mercury sphygmomanometer (Baumanometer; W.A. Baum Co., Copiague, NY, USA). After 3 measurements at interval of 5 min, the average of measured value was used for analytics. Hypertension was stated as a systolic pressure was 140 mmHg or higher or diastolic pressure was 90 mmHg or higher, or the use of prescribed anti-hypertensive medication. Prehypertension was defined as a systolic pressure of 120–139 mmHg or a diastolic pressure of 80–89 mmHg^45^.

Blood samples were taken after fasting for 10–12 h. The fasting glucose, glycated hemoglobin, triglyceride and total cholesterol were taken by Hitachi automatic analyzer 7600 (Hitachi, Ltd., Tokyo, Japan). All blood samples for analysis were properly processed, promptly refrigerated, and transported in cold storage to the Neodin Medical Institute (Seoul, Korea), a Korean Ministry of Health and Welfare-certified laboratory. Serum levels of vitamin A are measured by isocratic high performance liquid chromatography with detection at three different wavelengths. Serum is mixed with an ethanol solutionof the internal standards. The analytes are extracted into hexane, which is removed under vacuum. The extract is redissolved in ethanol; an equal volume of acetonitrile is then added. The extract is filtered to remove insoluble material. An aliquot of the filtrate is injected onto a C18 reversed-phase column and eluted with a 50% ethanol:50% acetonitrile solution containing 100 μL of diethylamine per liter. Chromatograms are recorded. Quantitation is accomplished by comparing the peak height of the analyte in the unknown with the peak height of a known amount of the same analyte in a standard solution. A correction based on the peak height of an internal standard is used. The prevalence of diabetes was divided into three stages. ‘Normal’ was defined as case that fasting glucose was 100 or less, and ‘fasting blood sugar disorder’ was defined as case that fasting glucose was 100 or more and 125 or less. ‘Diabetes’ was defined as case that fasting glucose was 126 or higher, taking diabetes drugs, or injecting insulin. Vitamin A levels in the blood are divided into four levels with equal participant number depending on the severity; Level 1: Blood vitamin A < 0.41, Level 2 : 0.41 ≤ Blood vitamin A < 0.51, Level 3 : 0.51 ≤ Blood vitamin A < 0.64, Level 4 : Blood vitamin A ≥ 0.64. The fundus photography was measured using a fundus optical instrument (VISUCAM 224, Carl Zeiss Meditec AG, Jena, Germany). Diabetic retinopathy was diagnosed if lesions defined by the Early Treatment for Diabetic Retinopathy Study were existent: these were characteristic lesions as hemorrhage, microaneurysm, hard exudate, intraretinal microvascular abnormalities, cotton wool spots, new vessels, venous beading and cotton wool spots^46^.

This study used complex sampling design for analysis. Complex sample surveys involve the identification and data collection of a sample of population units via multiple stages or phases of identification and selection. Logistic regression analysis was conducted to assess the relationship between blood vitamin A and diabetic retinopathy. The value that is not adjusted is defined as 'Crude'. ‘Model 1’ is an analysis after adjusting the variables of age and sex. 'Model 2' is a value analyzed after adjusting confounding variables including age, sex, smoking status, cholesterol, diabetes prevalence period, glycated hemoglobin levels, and high blood pressure. Statistical analysis was taken through SPSS (ver. 18.0; SPSS, Inc., Chicago, IL, USA), and was defined as statistically significant when P value was less than 0.05.

## Data Availability

The datasets generated during and/or analyzed during the current study are available from the corresponding author on reasonable request.
